# Development of an Induced Pluripotent Stem Cell-Based Liver-on-a-Chip Assessed with an Alzheimer’s Disease Drug

**DOI:** 10.1021/acsbiomaterials.3c00346

**Published:** 2023-06-15

**Authors:** Francesca Fanizza, Lucia Boeri, Francesca Donnaloja, Simone Perottoni, Gianluigi Forloni, Carmen Giordano, Diego Albani

**Affiliations:** † Department of Chemistry, Materials and Chemical Engineering ’Giulio Natta’, Politecnico di Milano, Milan 20133, Italy; ‡ Department of Neuroscience, Istituto di Ricerche Farmacologiche Mario Negri IRCCS, Milan 20156, Italy

**Keywords:** organ-on-a-chip, liver, liver-on-a-chip, induced pluripotent stem cells, drug testing, donepezil

## Abstract

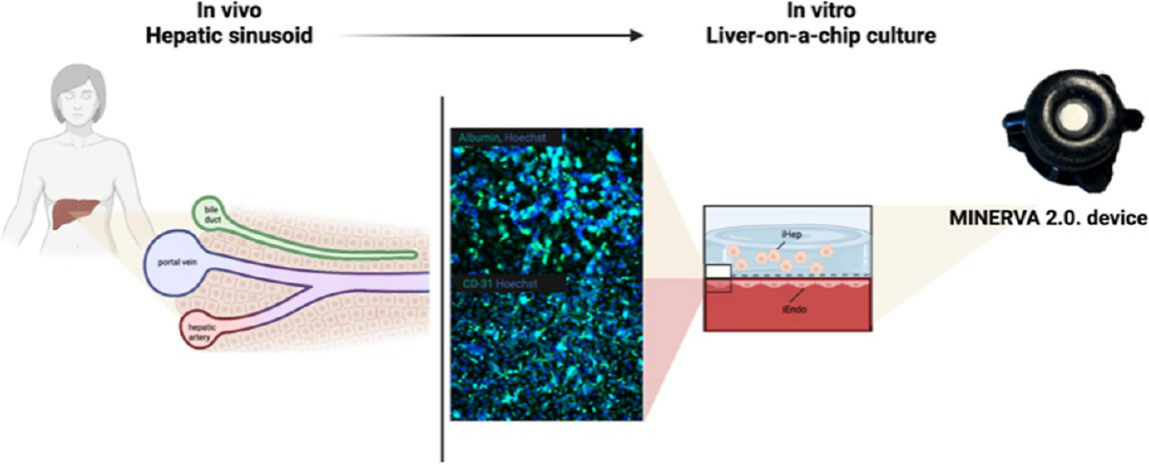

Liver-related drug metabolism is a key aspect of pharmacokinetics and possible toxicity. From this perspective, the availability of advanced in vitro models for drug testing is still an open need, also to the end of reducing the burden of in vivo experiments. In this scenario, organ-on-a-chip is gaining attention as it couples a state-of-the art in vitro approach to the recapitulation of key in vivo physiological features such as fluidodynamics and a tri-dimensional cytoarchitecture. We implemented a novel liver-on-a-chip (LoC) device based on an innovative dynamic device (MINERVA 2.0) where functional hepatocytes (iHep) have been encapsulated into a 3D hydrogel matrix interfaced through a porous membrane with endothelial cells (iEndo)]. Both lines were derived from human-induced pluripotent stem cells (iPSCs), and the LoC was functionally assessed with donepezil, a drug approved for Alzheimer’s disease therapy. The presence of iEndo and a 3D microenvironment enhanced the expression of liver-specific physiologic functions as in iHep, after 7 day perfusion, we noticed an increase of albumin, urea production, and cytochrome CYP3A4 expression compared to the iHep static culture. In particular, for donepezil kinetics, a computational fluid dynamic study conducted to assess the amount of donepezil diffused into the LoC indicated that the molecule should be able to pass through the iEndo and reach the target iHep construct. Then, we performed experiments of donepezil kinetics that confirmed the numerical simulations. Overall, our iPSC-based LoC reproduced the in vivo physiological microenvironment of the liver and was suitable for potential hepatotoxic screening studies.

## Introduction

Drug development is a long and costly process characterized by an overall failure rate of 90% in clinical trials.^1−3^

The organ-on-a-chip (OoC) technology, in line with the 3R principle of reducing, refining, and replacing in vivo testing, could have an impact on the drug development pipeline by providing improvements to current human in vitro models thanks to the OoC potential to better reflect human physiology.^4^

OoC is a biomimetic system that recapitulates the structural and functional characteristics of a human tissue.^5^ By integrating a fluid flow, OoC can provide mechanical stimuli and biochemical concentration gradients crucial for both cell growth and functionality and a reliable prediction of drug pharmacokinetic profile and toxicity.^4^ Moreover, the connection of multiple OoC devices into body-on-a-chip platforms allows the preliminary analysis of organ cross-talks and integrated responses to drug administration.^6^ The cultivation of differentiated induced pluripotent stem cells (iPSCs) into the OoC provides a valuable tool to develop patient-specific drug screening models, having also the potential to better predict side effects and thus contributing to reduce the percentage of drug failure in clinical trials,^7^ also in a personalized medicine perspective.

Drug-induced liver injury is an adverse event that frequently leads to drug failure in trials and withdrawal from the market.^8^

Thus, in recent years, several models of iPSC-based liver-on-a-chip (LoC) have been developed to summarize the physiology of the liver and to be applied in drug screening studies. As it is widely acknowledged, the liver is essential for the regulation of amino acids, carbohydrates, and fatty acids, the synthesis of proteins such as albumin and bile acids, as well as for the metabolism of endogenous substrates and exogenous compounds.^9^

In the present work, we propose an innovative LoC based on a new millifluidic OoC we developed, named MINERVA 2.0, hosting iPSC-derived hepatocytes (iHep) encapsulated into a collagen-polyethylene glycol matrix^10^ and cultured interconnected with a 2D layer of iPSC-derived endothelial cells (iEndo) to recapitulate the key features of the liver sinusoid. To assess its potential to be used for drug screening purposes, our LoC has been assessed with the drug donepezil approved for Alzheimer’s disease (AD).^11^

## Experimental Section

### Millifluidic Device

MINERVA 2.0 is a 3D printed in Nylon millifluidic OoC device compatible with commercial cell culture inserts.

MINERVA 2.0. was sterilized by UV rays (SafeMate cabinet) for 10 h or with hydrogen peroxide (V-PRO 60 Low Temperature Sterilization System).

### Cell Culture and Maintenance

#### iPSC-Derived Liver Cells

Cryopreserved iCell Endothelial Cells (iEndo) and Hepatocytes (iCell Hepatocytes 2.0) were purchased from Fujifilm Cellular Dynamics, Inc. (CDI).

iEndo were plated according to the manufacturer’s protocol. iEndo were thawed at 37 °C in a water bath for 3 min and contents immediately transferred into 10 mL of 37 °C endothelial medium composed of VascuLife VEGF Medium (Lifeline Cell Technologies, Frederick, MD) supplemented with the complete growth factors per the kits. Here, only 10 mL of the glutamine solution was added per 500 mL of media and 50 mL of the CDI-provided supplement replaced the VascuLife FBS component.

After centrifuging the cell suspension at 200 g for 5 min, the cell pellet was resuspended in fresh endothelial medium to obtain a desired cell plating density.

iCell Hepatocytes 2.0 (iHep) were plated according to the manufacturer’s protocol. iHep were thawed at 37 °C in a water bath for 3 min and contents immediately transferred into 10 mL of a 37 °C plating medium composed of 75 mL of RPMI 1640 (Thermo Fisher Scientific) supplemented with 1.5 mL of B27 supplement 50X (Thermo Fisher Scientific), 150 μL of Oncostatin M 10 μg/mL (Merck), 1.5 μL of Dexamethasone 5 mM (Thermo Fisher Scientific), 37.5 μL of Gentamicin 50 mg/mL (Thermo Fisher Scientific), and 1.5 mL of iCell Hepatocytes 2.0 medium supplement (CDI).

After centrifuging the cell suspension at 200 g for 3 min, the cell pellet was resuspended in fresh plating medium to obtain a desired cell plating density.

#### Transwell-like Co-Culture System

iHep and iEndo were co-cultured in commercial 12-well Transwell inserts (Greiner Bio-One, 665641) having a PET membrane with a pore diameter of 0.4 μm, density of 2 × 10^6^ pores/cm^2^. The iHep to iEndo cell ratio (4:1) was close to what was observed in vivo.^12^

iEndo were plated on the bottom side of the Transwell-like insert membrane pre-coated with Fibronectin (Promocell) according to the manufacturer’s protocol. Afterward, the 12-well plates were placed in the incubator upside down to allow the cells to firmly attach to the microporous membrane. After 1 h, the 12-well plates were turned over and replenished with new fresh medium.

Finally, iHep were seeded onto the top side of the Transwell -like insert membrane pre-coated with Collagen I (Sigma-Aldrich) according to the manufacturer’s protocol. The resultant Transwell plate was then incubated at 37 °C in 5% CO_2_. Afterward, the 12-well plates were placed in the incubator, and after 4 h, a complete medium exchange was performed with fresh plating medium.

Hepatocyte plating medium change was performed on a daily basis until day 5. After day 5, the plating medium was replaced by a maintenance medium, which contained all the supplements in the plating medium with the exception of Oncostatin M. The endothelial medium was changed every two days.

We examined two liver cell models:

- 2D model w/ or w/o iEndo: 3 × 10^5^ iHep were plated on the upper side of the Transwell -like insert membrane, while 5 × 10^4^ iEndo were plated on the lower side of the Transwell -like insert membrane.
- 3D liver w/ iEndo: 3 × 10^5^ iHep were mixed with a polymeric solution and plated on the upper side of the Transwell -like insert membrane, while 5 × 10^4^ iEndo were plated on the lower side of the Transwell -like insert membrane. The polymeric solution composed of type I collagen (COLL) (Sigma-Aldrich) and poly(ethylene)glycol (PEG) with *M*_w_ = 2000 Da was prepared as described in a previously published paper.^10^ The COLL–PEG gel loaded with cells was 1.5 mm high.

### Numerical Evaluation of the Millifluidic Device with the 3D Cell Model

To select the optimal flow rate for the dynamic culture of liver cells in the millifluidic-on-a-chip device, a computational simulation was performed with COMSOL Multiphysics, release 5.6. The geometries of the models for the internal space of the culture hemi-chambers were obtained with Solidworks software, release 2019. In the internal space of the chambers, two separate flow pathways were considered for simulations. The first flow enters from the inlet of the lower chamber, perfuses the endothelial cells culture, and exits from the facing outlet, while the second flow enters from the inlet of the upper chamber, perfuses the hepatoctes culture, and exits from the facing outlet.

The simulations were performed with perfusion in the counter-current configuration. The velocity field, shear stress, and oxygen distribution were computed in both chambers using “free and porous media flow” and “transport of diluted species” physics.

For the reaction terms, we assumed a homogeneous cell distribution within the hydrogel.

We determined the fluid velocity vector, u, using the Brinkman equation in stationary conditions, eq 1, suitable for porous media and the mass balance, eq 2
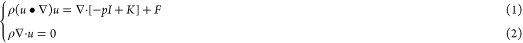
where ρ is the fluid density, *u* is the velocity vector, *p* is the fluid pressure, *I* is the identity matrix, *K* is the permeability tensor, and *F* is the volume force vector. The permeability vector for porous media is defined in eq 3
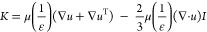
3where μ is the dynamic viscosity and ε is the porosity.

The oxygen distribution was estimated with the transport equation for diffusion and convection eq 4
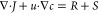
4with *J* being the mass flux vector, *c* the concentration, *R* the oxygen volumetric consumption rate, and *S* the mass source. The mass flux vector is defined by Fick’s law eq 5
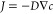
5with *D* being the oxygen diffusion coefficient in the fluid.

For the oxygen consumption rate, it was assumed that the reaction term *R* was a function of the local oxygen concentration according to the Michaelis–Menten kinetics eq 6
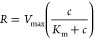
6where *V*_max_ is the maximum molar consumption rate, *c* is the local oxygen concentration, and *K*_m_ is the Michaelis–Menten constant. The Michaelis–Menten constant corresponds to the oxygen concentration at which the consumption is half of the *V*_max_.

Shear stress was obtained with the following formula, eq 7
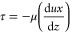
7where *ux* is the velocity component vector in the *x*-direction (parallel to the perfusion direction) and *z* is the direction perpendicular to the flow direction.

To run the simulation, we chose the “Fine” element size for mesh building.

All the characteristic parameters are summarized in Table 1 in the Supporting Information.

### Dynamic Culture of the 3D Cell Model in the Millifluidic Device

After 5 days in static conditions, the Transwell -like inserts hosting the cell-based 3D model were assembled into the MINERVA 2.0. device and perfused by a peristaltic pump (Longer Precision Pump Co., Ltd.) in counter-current configuration at a flow rate of 30 μL/min (the same flow rates applied for CFD simulations). A total of 8 mL of hepatocyte maintenance medium for the upper chamber and 8 mL of the endothelial medium for the lower chamber were used to perfuse each chip by recirculation for 7 days.

Correspondingly, the static culture was set as a control group, and the media were changed every two days. The dynamic culture was maintained at 37 °C in a humidified atmosphere containing 5% CO_2_.

After 7 days of dynamic culture, the devices were disassembled from the circuit by opening the connectors. Sample media were harvested from the reservoirs and Transwell -like inserts were placed within 12-well plates for the analysis.

### Biological Assays

#### Cell Viability Assessment

To investigate the cell metabolic activity, the MTS metabolic activity test was performed using the kit CellTiter 96 Aqueous One Solution Cell Proliferation Assay (Promega). To assess the cytotoxicity in the dynamic condition, the CyQUANT LDH Cytotoxicity Assay Kit (Invitrogen) was used to detect the amount of LDH in the medium with a colorimetric reaction.

#### Albumin and Urea Production

Albumin and urea production was analyzed to assess the liver-specific functions by collecting the medium after 7 days of culture. Albumin concentration in the medium was determined by an enzyme-linked immunosorbent assay (ELISA) kit (Bethyl Laboratories). Urea concentration was evaluated by using a urea assay kit (Sigma-Aldrich) following the manufacturer’s protocol.

Levels of albumin and urea secretion were quantified and normalized per hour to the sample volume and seeded cells.

All collected samples of the cell supernatant were kept frozen at −80 °C prior to performing the assays. Samples were thawed to room temperature and prepared according to the manufacturer’s protocol.

#### Immunofluorescence Staining

3D cultures within Transwell -like inserts were fixed by adding 4% paraformaldehyde for 24 h at RT. The fixative was removed by TBS-t 1X rinsing. A blocking buffer was added (50 mM Tris, 0.1% Tween-20, 0.3 M glycine, 4% NGS, 1% BSA, and 1 mg/mL gelatin in TBS-t) for 4–6 h and then replaced with TBS-t washing. Blocking buffer containing an anti-human albumin antibody (1:7500; Cedarlane) was incubated for 24 h to label iHep and then washed with TBS-t. The Alexa Fluor 488 anti-mouse IgG secondary antibody (1:500; Invitrogen; Life Technologies) was used to visualize albumin. Cultures were counterstained with Hoechst (Invitrogen) to visualize nuclei. The 3D model was observed directly in the Transwell -like insert with a confocal microscope Olympus FV10i.

2D cultures within Transwell -like inserts were fixed by adding 4% paraformaldehyde for 30 min. The fixative was removed by PBS rinsing, and the fixed cultures were treated for 15 min with 0.3% TritonX-100 in PBS permeation solution. The permeation buffer was replaced with PBS washing followed by 20 min with blocking buffer (4% BSA, 0.25% Triton X-100 in PBS). The blocking buffer contained anti-human CD-31 (1:100, Invitrogen) and anti-human zonulin-1 (1:100, Invitrogen)-labeled iEndo. The Alexa Fluor 647 anti-rabbit IgG secondary antibody (1:500; Invitrogen; Life Technologies) was used to visualize zonulin-1, while the Alexa Fluor 488 anti-mouse IgG secondary antibody (1:500; Invitrogen; Life Technologies) was used to visualize CD-31. Cultures were counterstained with Hoechst (Invitrogen) to visualize nuclei. At the end of the protocol, the Transwell-like insert membranes were cut with a scalpel and placed on a microscope slide for visualization with a confocal microscope Olympus FV10i.

#### Western Blot Analysis

On day 7, the samples were lysed at 4 °C with lysis buffer for 1 h and stored at −80 °C. Before Western blotting, we centrifuged the lysates at 4 °C for 10 min at 13,000 rpm.

We evaluated the protein content with a bicinchoninic acid protein assay kit (PierceTM; Thermo Fisher Scientific) and loaded 20 μg of protein in an 8% sodium dodecyl sulfate polyacrylamide gel electrophoresis system.

We transferred the electrophoresis gel to a nitrocellulose membrane (BioRad Laboratories). We incubated the membrane with albumin monoclonal primary antibody (Cederlane, 1:1000) or *p*-glycoprotein (*p*-gp) monoclonal antibody (Thermofisher, 1:200) overnight at 4 °C with horseradish peroxidase-conjugated antimouse IgG antibody (Jackson ImmunoResearch, 1:15,000) and then used enhanced chemiluminescence as the detection system (Millipore). We developed the immunoreactive bands with a Firereader V10 PLUS 26M Imaging system (Uvitec Ltd.) and quantified by ImageJ software. The obtained values were then normalized on the GAPDH signal coming from the same samples.

#### Real-Time PCR

Cultured cells were lysed in QIAzol Lysis Reagent (Qiagen) and stored at −80 °C before RNA extraction. Total RNA was extracted from cell lysates using a miRNeasy Mini Kit (Qiagen) and reverse-transcribed to cDNA using a High-Capacity cDNA Reverse Transcription Kit (Applied Biosystems). qPCR was performed on a MasterCycler EP Gradient S (Eppendorf) using TaqMan gene expression assays (Applied Biosystems) and CYP3A4 (Hs00604506, Applied Biosystems) and HNF4-alpha probes (Hs00230853_m1, Applied Biosystems).

Expression levels were normalized to β-actin (Hs99999903, Applied Biosystems). Gene expression levels were calculated using the delta–delta CT method relative to the level in iHeps 2D w/o EC or to the level in iHeps in the static condition.

### Drug Diffusion Study

#### Donepezil Hydrochloride

Donepezil hydrochloride (cat. #D6821, Merck) was dissolved in dimethyl sulfoxide (DMSO) to a stock concentration of 10 mM and diluted in iEndo medium to a 200 μM final concentration.

#### In Vitro Drug Diffusion Experiment

The diffusion mechanism of donepezil through the COLL–PEG hydrogel was investigated by adding the donepezil solution into the donor chamber of the Transwell -like insert hosting the hydrogel. The amount of donepezil diffused through the hydrogel was quantified by spectroscopy of the samples taken from the medium in the acceptor chamber of the insert at predetermined time intervals.

The analysis was made by means of a fluorescence microplate reader (Tecan Infinite M200) at a 233 nm excitation wavelength and a 400 nm emission wavelength. To simulate the perfect release conditions, the medium was replaced by fresh medium after each sampling. The subsequent results were normalized with the primary amount of drug load in the donor chamber and expressed in percentage.

#### Mathematical Modeling

To find out the mechanism of drug release from the hydrogel, the non-linear regression model Korsmeyer–Peppas was used. This model has been described with the equation
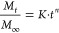
8In this equation, *M*_*t*_/*M*_*∞*_ represents the fraction of drug released at time *t, K* is the release rate constant (dimension of time^–1^), and *n* is the transport exponent (dimensionless). In this model, the value of *n* characterizes the release mechanism of the drug. For the case of hydrogels, 0.5 ≤ *n* corresponds to a Fickian diffusion mechanism, 0.5 < *n* < 1 to non-Fickian transport, *n* = 0.89 to Case II (relaxational) transport, and *n* > 1 to super case II transport. To study the release kinetics, data obtained from in vitro drug release studies were plotted as log cumulative percentage drug release versus log time.

Drug transport constants (*K*) and transport exponents (*n*) of the COLL–PEG hydrogel were determined by fitting of the in vitro diffusion data to the Korsmeyer–Peppas equation (see eq 8) using the add-in DDSolver program (China Pharmaceutical University, Nanjing, China). Microsoft Office Excel (Microsoft Corporation, Redmond, USA) was used as built-in module of the DDSolver.

#### Drug Diffusion Coefficient D Determination

The drug diffusion coefficient through the COLL–PEG hydrogel hosted in a Transwell -like insert can be calculated with the Fick’s Law for diffusion for a diffusion cell
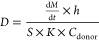
9where d*M*/d*t* is the diffusion rate, *h* is the height of the COLL–PEG hydrogel, *S* is the area of the Transwell -like insert, *K* is the partition coefficient, and *C*_donor_ is the drug concentration in the donor chamber of the Transwell -like insert.

#### Computational Simulation

To estimate the amount of donepezil diffused though the hydrogel in the millifluidic-on-a-chip device, a computational simulation was performed with COMSOL Multiphysics, release 5.6.

In the internal space of the chambers, two separate flow pathways were considered for simulations. The first flow enters from the inlet of the lower chamber, perfuses the endothelial cells culture, and exits from the facing outlet. The second flow enters from the inlet of the upper chamber and exits from the outlet.

The simulations were performed with perfusion in counter-current configuration. The donepezil distribution was computed in both chambers using “free and porous media flow” and “transport of diluted species” physics.

We determined the fluid velocity vector u using eqs 1 and 2 and the permeability vector for porous media with eq 3.

The donepezil distribution was estimated with the transport equation for diffusion and convection, eq 4, with a *J* mass flux vector, a c concentration, an *S* mass source, and no reaction term *R*. The mass flux vector is defined by the Fick’s law eq 5 in which *D* is the donepezil diffusion coefficient.

To run the simulation, we chose the “Fine” element size for mesh building. A time-dependent simulation was performed for *x*–*y* h with a *zh* time step. The concentration of the drug in the apical outflow was determined by placing a point probe and programming the probe to output the concentration of the drug.

Parameters used in this simulation are provided in Table 2 in the Supporting Information.

#### Donepezil Dosing within the LoC

Donepezil was dissolved into iEndo medium and perfused through the basal chamber of the millifluidic device, while the apical chamber hosting the COLL–PEG hydrogel was perfused with iHep medium at 30 μL min^–1^. Medium from the outlet of the apical chamber was collected at 6, 24, 48, and 72 h and analyzed through spectroscopy to quantify the amount of donepezil diffused.

The concentration of donepezil at the outlet of the apical chamber was normalized to the inlet concentration in the basal chamber. The injection of donepezil through the inlet of the basal chamber and the sampling from the outlet of the apical chamber occurred by means of a 3-way valve.

The dynamic culture was maintained at 37 °C in a humidified atmosphere containing 5% CO_2_.

After 72 h of dynamic culture, the devices were disassembled from the circuit by opening the connectors and the Transwell -like inserts were placed within 12-well plates for the analysis.

## Statistics

Each experiment was performed at least in triplicate and independently replicated two or three times. Results are reported as mean ± standard deviation (SD). We analyzed the data with GraphPad Prism software (GraphPad Software, Inc.). We used one-way analysis of variance (ANOVA) followed by Tukey’s multiple comparisons test and Mann–Whitney *U* test. We set the significance level at 0.05.

## Results

### MINERVA 2.0. Millifluidic Device

**Figure 1:**
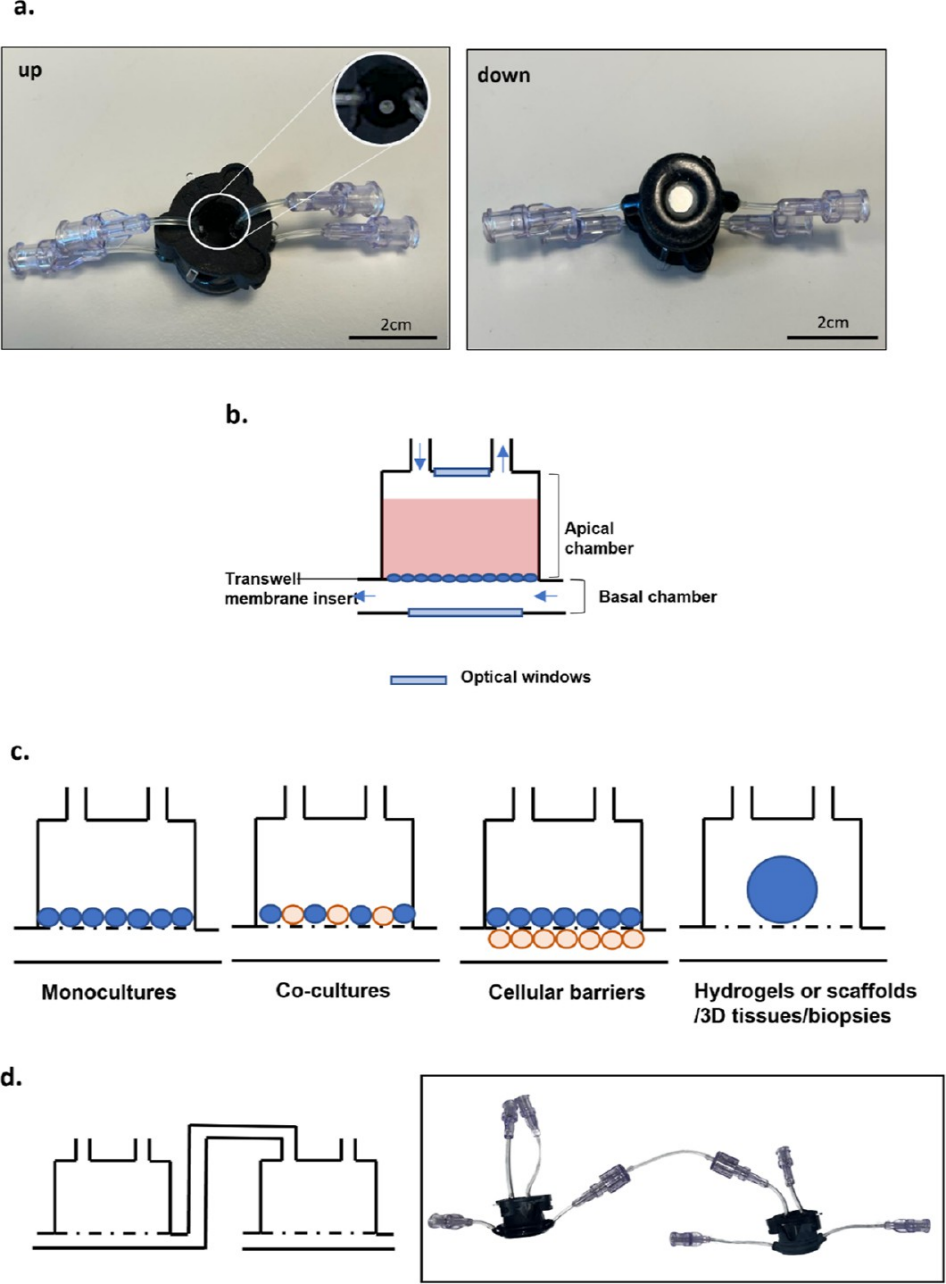
MINERVA 2.0. device. (a) Upper and lower view of the MINERVA 2.0 millifluidic device. Close-up of the optical window. (b) Schematic representation of the MINERVA 2.0. internal spaces. The arrows indicate the direction of the fluid flow. (c) Sketch of the MINERVA 2.0. cell culture configurations. (d) The luer-lock connection allows to serially connect the single units to build up multi-organ platforms.

Our innovative device named MINERVA 2.0 consists of two nylon 3D-printed components assembled with a snap-fit closure system and a 12-well Transwell-like insert (Figure 1). Inside the device, there are two hemi-chambers, apical and basal, interfaced through a porous membrane of the Transwell-like insert. The apical chamber is 2 mm high, while the basal is 0.5 mm. A reliable seal is ensured by the use of two O-rings on the apical and basal chambers.

A transparent glass slide is mounted on the apical and basal chambers to allow for monitoring cell culture by optical or confocal microscopy. The independent perfusion of the apical chamber is counter current with the basal one (Figure 1b). To implement a multi-OoC platform using MINERVA 2.0, our device hosts luer-lock connectors coupled to millifluidic channels with diameters of 0.5–1 mm (Figure 1d).

### Development of an iPSC-Based 3D Liver Model

**Figure 2:**
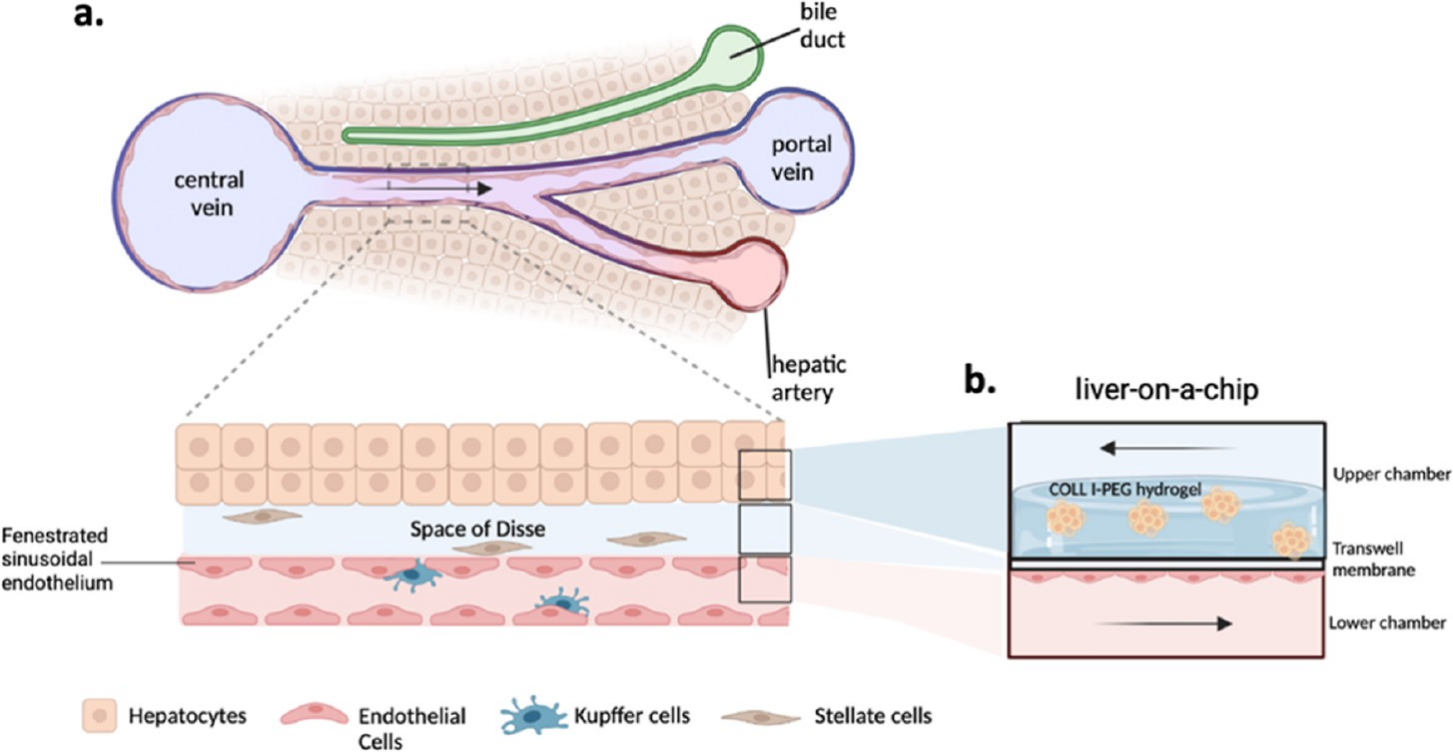
a) Cellular composition and anatomical structure of the liver sinusoid, the functional unit of the liver.^14^ (b) Schematic representation of our LoC hosting the key components of the liver sinusoid (cross section). Created with Biorender.com.

To recall the features of a human liver sinusoid structure (Figure 2a), we designed our liver model by exploiting the two adjacent chambers separated by the Transwell -like insert porous membrane proper of the MINERVA 2.0 device. In the upper chamber, iHep were seeded on the top side of the membrane and encapsulated into a COLL–PEG hydrogel, while in the lower chamber, iEndo were seeded on the bottom side of the membrane (Figure 2b). In this configuration, the passive diffusion of molecules such as nutrients and oxygen occurs through the membrane, representing the Space of Disse, the perisinusoidal area between the hepatocytes, and endothelial cells.^13^

**Figure 3:**
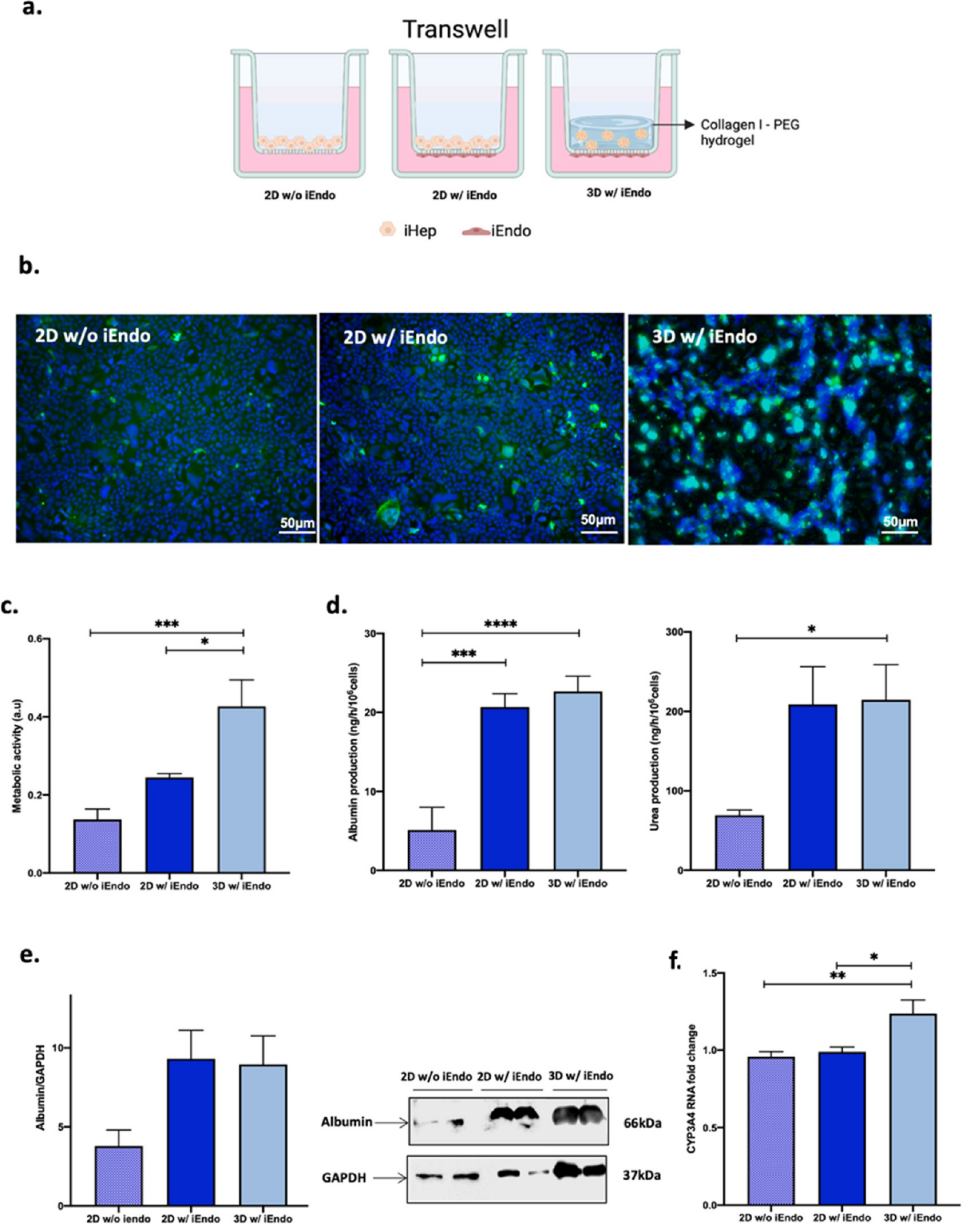
(a) The schematic shows the disposition of the different cell types into the Transwell-like system. (b) *Z*-stack projection of immunofluorescence confocal microscopy images of iHep after 7 days of culture. Green = albumin and blue = Hoechst nuclear staining. Magnification: 10X. (c) Metabolic activity of iHep. (d) Albumin production and urea synthesis by iHep. (e) Western blot of albumin in iHep. (f) Relative mRNA expression of CYP3A4 of iHep. One-way ANOVA, Tukey’s multiple comparison post hoc test ns = *p* > 0.05, * = *p* < 0.05; ** = *p* < 0.01; *** = *p* < 0.001; **** = *p* < 0.0001.

To investigate the modulation of the 3D environment and iEndo on iHep behavior, the 3D liver model described above (3D w/iEndo) was compared with two conditions: a 2D model of iHep interfaced with iEndo (2D w/iEndo) and a 2D model of iHep alone (2D w/o iEndo) (Figure 3a). At 7 days from maturation, iHep in 2D w/ and w/o iEndo displayed an adherent monolayer, while in 3D w/ iEndo displaced along the height of the gel (Figure 3b).

iHep in 2D or 3D conditions appeared viable and metabolically active as shown in Figure 3b,c. An increased metabolic activity of iHep encapsulated into the hydrogel with respect to the 2D conditions was also observed. Moreover, the functionality of iHep was evaluated by assessing the levels of key hepatic function biomarkers, such as albumin and urea (Figure 3d). Albumin and urea levels in culture media of iHep in 3D w/ iEndo were comparable with those in 2D w/ iEndo and significantly higher with respect to 2D w/o iEndo.

Moreover, there was no difference in albumin protein expression between the three conditions (Figure 3e).

In addition, the mRNA expression of CYP3A4, the most abundant cytochrome P450 enzyme involved in drug metabolism,^15^ indicated a significantly higher detoxification ability of iHep in the 3D condition, thus indicating the 3D w/ iEndo model as the optimal one for further studies (Figure 3f).

### Numerical Evaluation of the 3D Liver Model inside the Millifluidic Device

**Figure 4:**
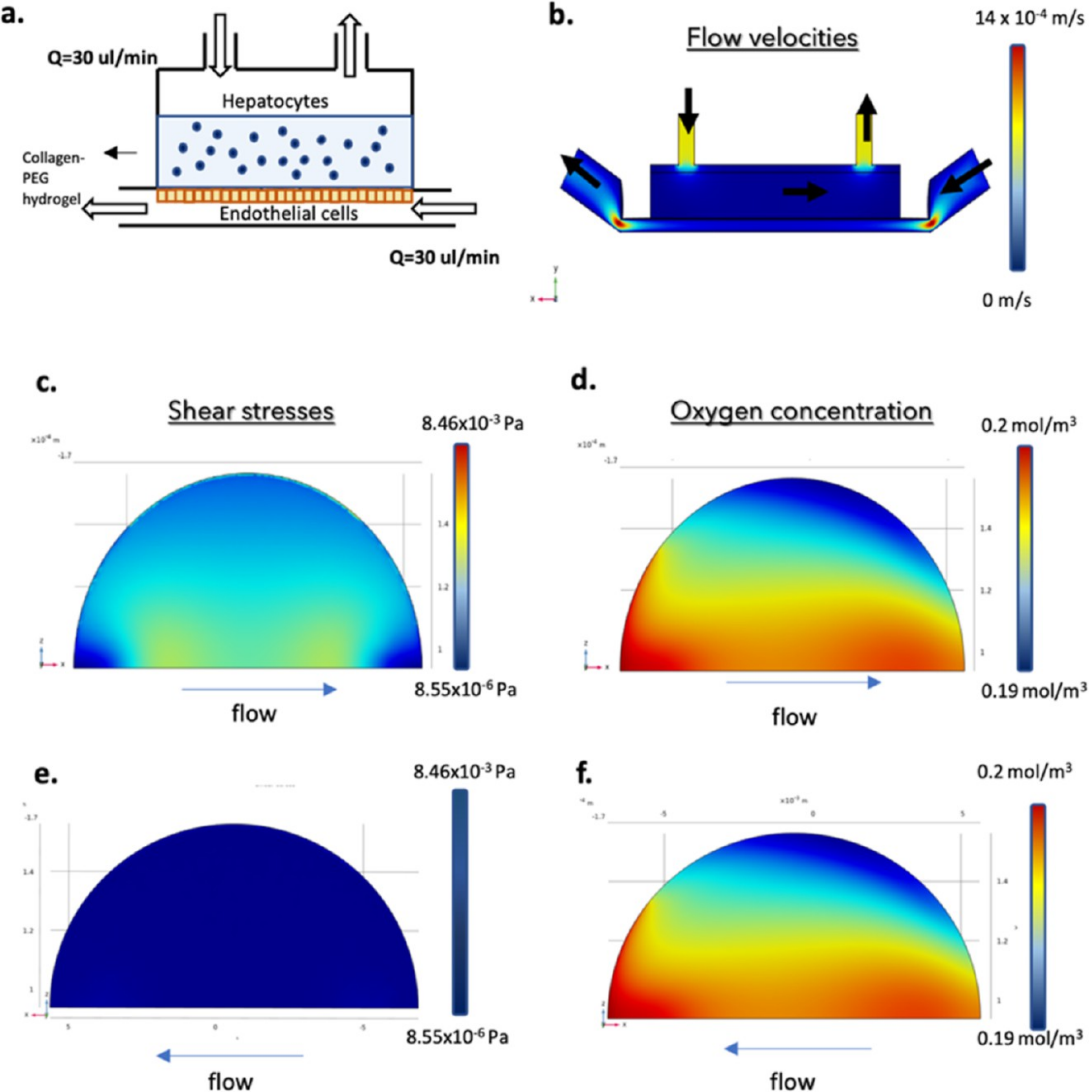
Computational fluid dynamic simulation prodromal to dynamic culturing of our LoC. (a) Lateral view of the geometric model. (b) Flow distribution and magnitude velocity (side view). (c) Shear stress on the upper side (top view) and (e) lower side (bottom view) of the porous membrane separating the two culture chambers of the MINERVA 2.0 device. (d) Oxygen concentration on the upper side (top view) and (f) lower side (bottom view) of the same membrane.

Once the biological features of our liver model in static condition were evaluated, we moved on to the dynamic culturing in MINERVA 2.0. First, we ran a numerical evaluation with the aim of assessing the optimal flow rate for the dynamic culture and the oxygen consumption and shear stresses profile of the iHep and iEndo into the LoC (Figure 4a).

Three different flow rates (10, 30, and 60 μL/min) were simulated in order to select the optimal one (data not shown). From the analyses of mean and maximum velocities and of maximum shear stress at the membrane symmetry plane, the flow rate of 30 μL/min turned out to be optimal to avoid the development of high velocities in the chambers that could detach cells from the membrane while allowing the development of low shear stresses.

In particular, with a flow rate of 30 μL/min, we simulated flow velocities in both chambers of the LoC that were within a physiologic range^16^ (Figure 4b). In detail, into the upper chamber through the hydrogel hosting the iHep, an interstitial fluid flow of approximately 1.5 × 10^–3^ mm/s was generated, while in the lower chamber, the flow ranged from 0.54 to 1.4 mm/s.

The numerical simulation showed adequate shear stresses (range 0.01–0.03 mPa) (Figure 4c) and oxygen consumptions (range 0.18–0.2 mol/m^3^) (Figure 4d) experienced by the iHep within the hydrogel under perfusion. These results were compared to the requirements of shear stress and oxygen concentration found in the literature. In particular, shear stress should be lower than 0.2 Pa for hepatocytes^17^ to avoid cell sufferance, while the oxygen concentration should be higher than 0.021 mol/m^3^.^18^ Moreover, the results showed that the shear stress (ranged 0.1–1 Pa) experienced by endothelial cells on the membrane of the lower chamber were slightly lower than the in vivo values for capillaries (Figure 4e).^19^

### Dynamic Culturing of the 3D Liver Model inside the Millifluidic Device

**Figure 5:**
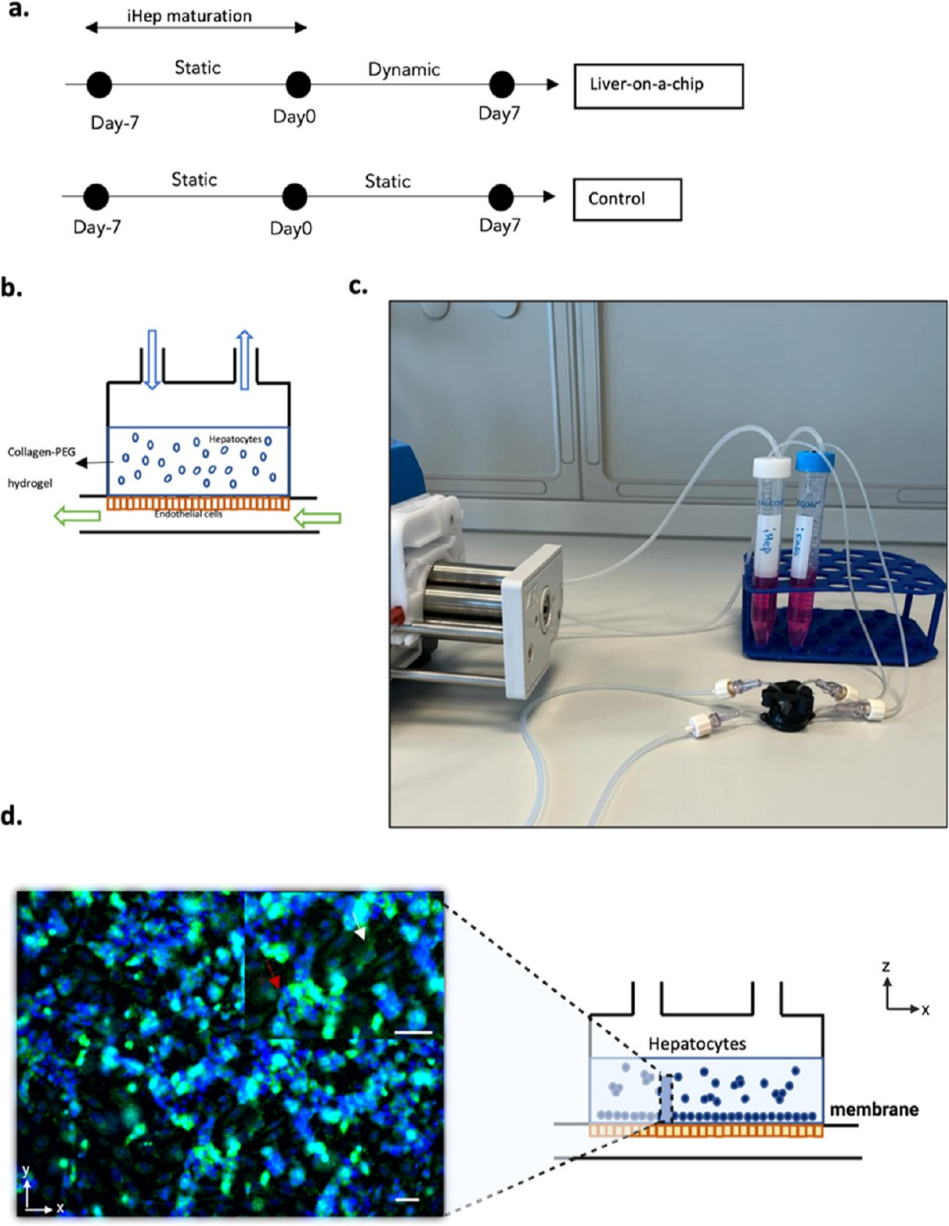
(a) Experimental timeline for the dynamic culture session. (b) Schematic representation of the LoC connected to the perfusion system. (c) Lateral view of the MINERVA 2.0 millifluidic device connected to the perfusion system. (d) *Z*-stack projection of immunofluorescence confocal microscopy images of iHep in the COLL–PEG gel after 7 days inside the MINERVA 2.0 device. Green = albumin and blue = Hoechst nuclear staining. Magnification: 10X. Scale bar: 20 μm. The white arrow points to the cells at the base of the hydrogel while the red arrow points to the cell above.

Based on the above-described computational results, we passed on to dynamic culturing of our LoC. At first, the 3D in vitro model was cultured in static conditions for 7 days to allow the maturation of the cell construct. Then, the inserts hosting the 3D w/ iEndo were assembled into the MINERVA 2.0. device and cultured under continuous perfusion with a flow rate of 30 μL/min in both chambers for further 7 days (Figure 5a–c). The same model but cultured in the static condition was used as a reference.

### Morphology and Function of iHep in the LoC

The three-dimensional spatial distribution of iHep within the COLL–PEG hydrogel was evaluated by immunofluorescence after 7 days of perfusion. The *Z*-stack projection image shows hepatocytes at the base of the hydrogel (pointed by the white arrow) and hepatocytes distributed along the height of the hydrogel (pointed by the red arrow) (Figure 5d).

**Figure 6:**
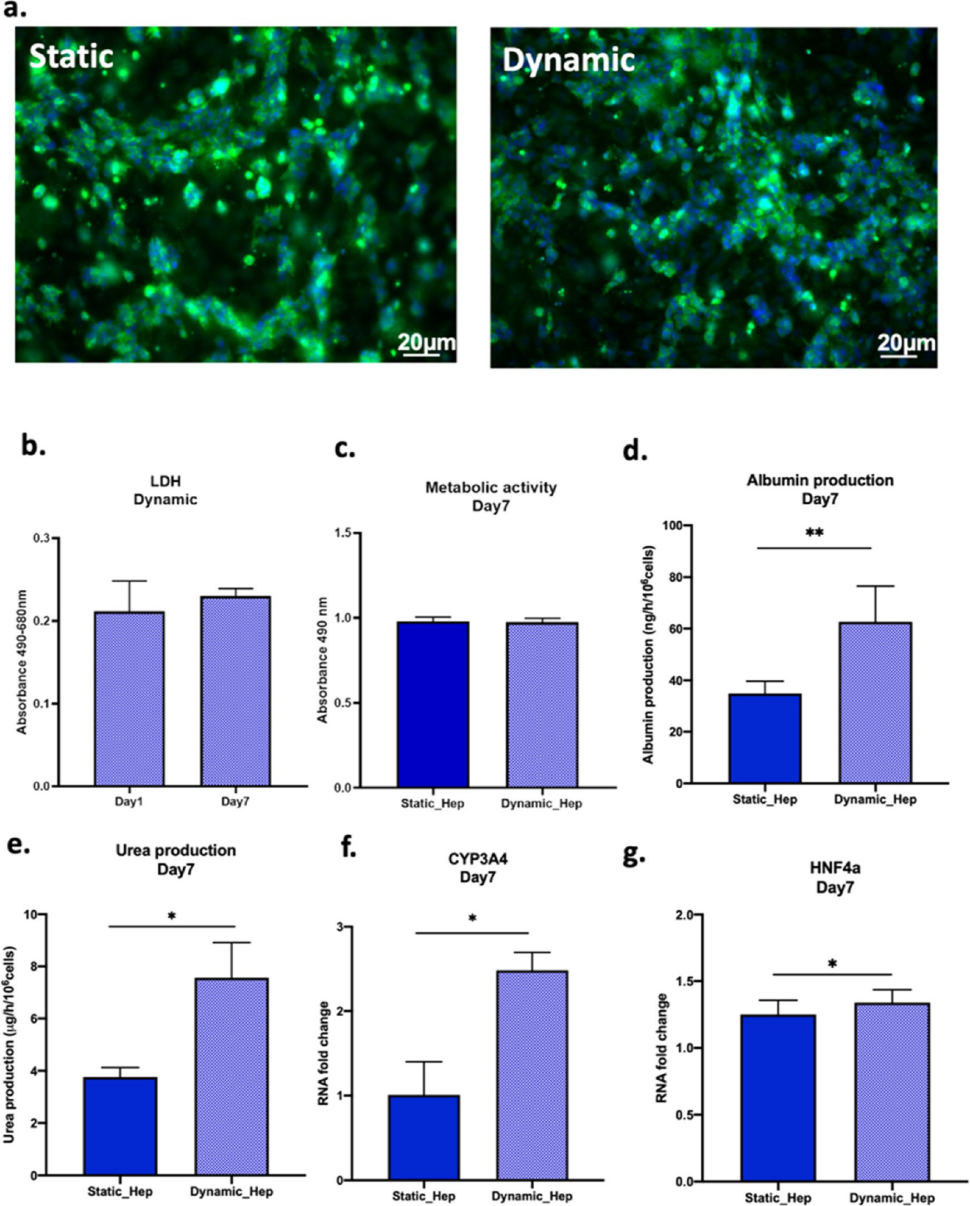
Cell layer maturation and functional analysis of iHep when cultured inside MINERVA 2.0. (a) *Z*-stack projection of immunofluorescence confocal microscopy images of iHep in the COLL–PEG gel in static (left) and perfused (right) conditions 7 days post maturation. Green = albumin and blue = Hoechst nuclear staining. Magnification: 10X. (b) Cytotoxicity at days 1 and 7 in perfused samples. (c) Metabolic activity. (d) Albumin production. (e) Urea synthesis. (f,g) mRNA relative expression of CYP3A4 and HNF4a in static and perfused samples 7 days post maturation. Mann–Whitney *U* test ns = *p* > 0.05, * = *p* < 0.05; ** = *p* < 0.01.

Results showed that the cells in the LoC remained viable throughout the experiments since no evident toxicity was detected in the culture (Figure 6a,b).

The spatial distribution of iHep in static and dynamic conditions does not differ. In addition, the TEER values are comparable (Supporting Information Figure S1) as well as the cell growth assessed by MTS assay (Figure 6c).

Moreover, the analysis of albumin and urea levels indicated an increased expression in the dynamic condition with respect to the static reference (Figure 6d,e).

Finally, the mRNA expression of the cytochrome CYP3A4 resulted in a higher culture of iHep inside the LoC, while the expression of HNF4-a (hepatocytes nuclear factor 4 alpha),^20^ a central regulator of hepatocyte differentiation, was comparable between the two conditions (Figure 6f,g).

### Morphology and Function of iEndo in the LoC

**Figure 7:**
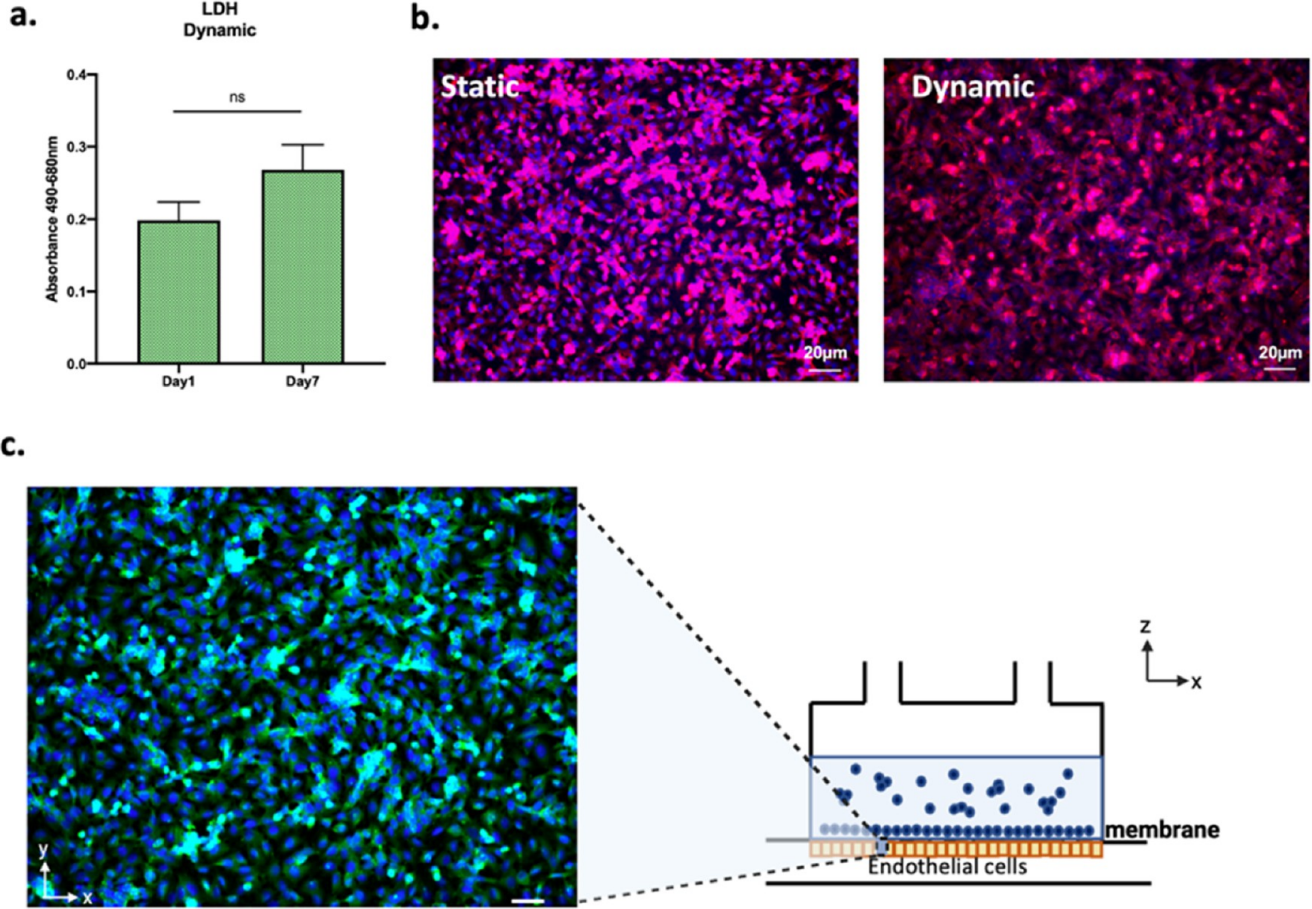
Cell layer maturation and viability of iEndo when cultured inside MINERVA 2.0. (a) Cytotoxicity at days 1 and 7 in perfused samples. Mann–Whitney *U* test, *p* > 0.05. (b) Immunofluorescence confocal microscopy images of iEndo cultured in static (left) and perfused (right) conditions 7 days post maturation. Red = zonulin-1 and blue = Hoechst nuclear staining. Magnification: 10X. Scale bar: 20 μm. (c) *Z*-stack projection of immunofluorescence confocal microscopy images of iEndo after 7 days of culture inside the millifluidic device. Green = CD-31 and blue = Hoechst nuclear staining. Magnification: 10X. Scale bar: 20 μm.

Endothelial cells were viable throughout the culture time as shown by the low levels of cytotoxicity and developed a strict monolayer in both static and dynamic cultures (Figure 7a,b).

Moreover, as evidenced by the immunofluorescence images, their endothelial layer aligned along the direction of the fluid flow(Figure 7b).

Finally, immunofluorescence labeling for CD-31, a marker of endothelial differentiation, showed that iEndo maintained their differentiated state after the dynamic culture (Figure 7c).

### Evaluation of Donepezil Transport and Toxicity into the LoC

In order to assess the suitability of our MINERVA 2.0-based LoC for drug studies, we evaluated its performance when exposed to donepezil, the most commonly prescribed drug for AD treatment.^21^

To this end, we had previously run a computational model to predict donepezil kinetics through the LoC after 72 h, followed by experimental confirmation.

### In Vitro Diffusion Study of Donepezil into the COLL–PEG Hydrogel

**Figure 8:**
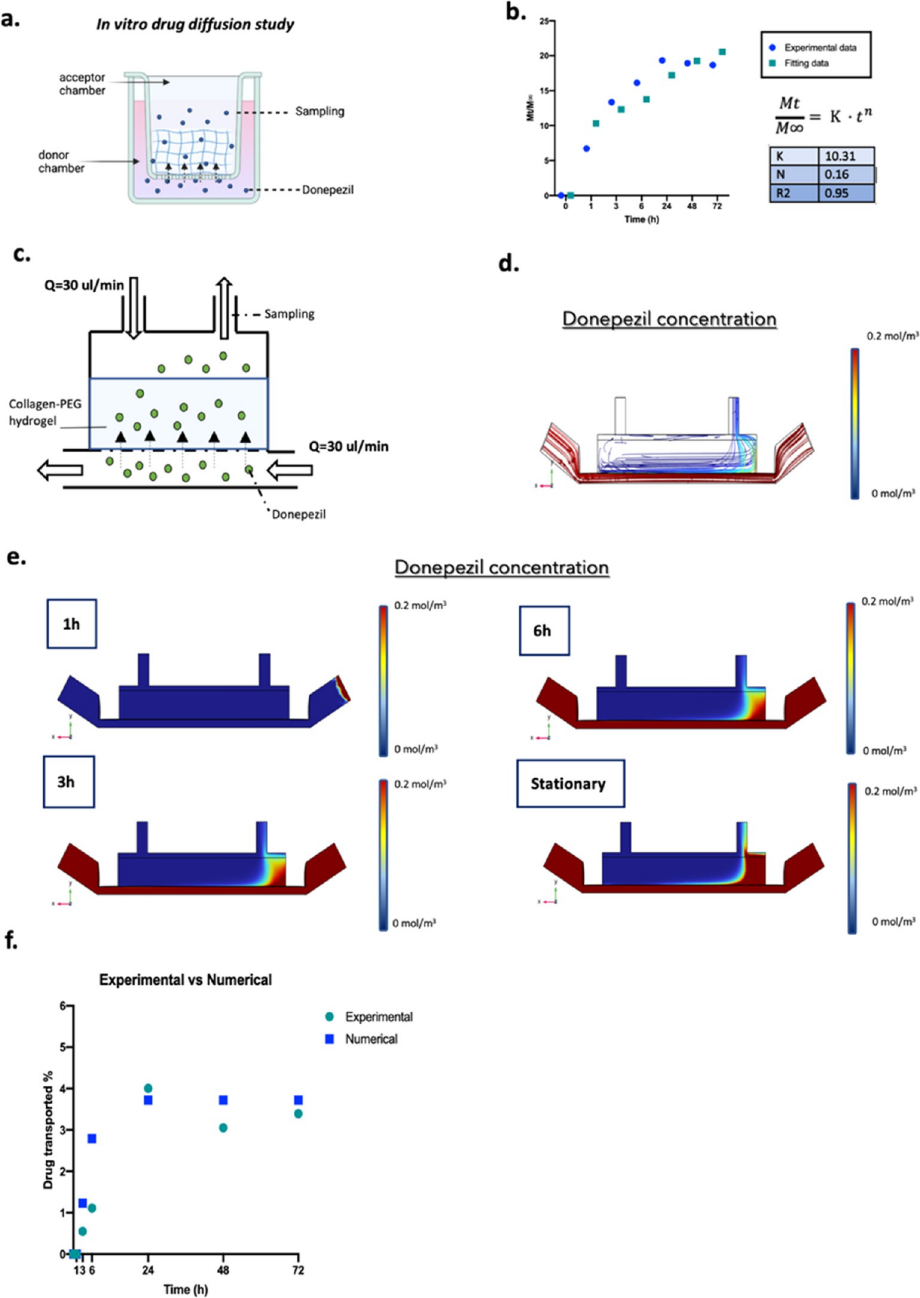
In vitro diffusion study of donepezil into MINERVA 2.0. (a) Experimental procedure, for details see the Experimental Section. (b) Drug diffusion profile through the hydrogel in the Transwell -like system. Fitting curve of the Korsmeyer–Peppas model. In the table are shown the Korsmeyer–Peppas parameters. (c) Side-view schematic of the millifluidic device hosting the hydrogel. (d) Streamline of donepezil concentration in the stationary condition. Side view. (e) Heat maps of drug concentration into the device after drug dosage. (f) Plot showing the experimentally determined and simulated donepezil transported in the outlet of the apical chamber over 72 h.

As previously described, our LoC was based on a 3D hydrogelic matrix embedding iHep in the upper chamber. Consequently, when exposed to donepezil, there might be an interaction with this matrix to be taken into account. To describe the release mechanism of donepezil through the COLL–PEG hydrogel, we performed a release kinetics and then applied the Korsmeyer–Peppas kinetic equation (Figure 8a) which predicts release mechanisms based on diffusion of liquid into the matrix, the swelling of the matrix, and dissolution of the matrix.

Figure 8b shows a plot of the cumulative amount of donepezil released from the hydrogel during time in which an initial burst is observed in the first 12 h of dissolution followed by a slow release, which tends to the asymptote of saturation concentration of the drug, which is 20% of the initial amount.

The results showed that the donepezil diffusion into the hydrogel fitted with the Korsmeyer–Peppas model as shown by the high coefficient of determination R2. The *n* and *K* values calculated from the slope of straight lines and intersections are shown in Figure 8b. The *n* value for the COLL–PEG hydrogel was found to be below 0.45; thus, donepezil was released into the hydrogel by simple Fickian diffusion. Moreover, the experimentally measured value of the partition coefficient of donepezil with the hydrogel was found to be close to 1, indicating that there was no absorption of the drug by the system.

### Numerical Modeling and Experimental Assessment of Donepezil Transport in the Millifluidic Device

Once it was assessed that the COLL-based matrix should not be involved in donepezil adsorption, we further implemented the computational model to incorporate all relevant factors to predict the donepezil distribution within the MINERVA 2.0 device (drug dosage, device geometry, temperature, and pressure) (Figure 8c). As for the donepezil input concentration, a clinically relevant dose of 200 μM did not induce hepatoxicity as confirmed by in vitro studies; thus, this was set as the initial concentration in our model.^22^

The results showed that after 6 h of drug treatment by perfusion under a flow rate of 30 μL/min, the drug at the outlet of the upper chamber that diffused through the basal chamber and through the hydrogel was 2.79% of the initial concentration (Figure 8d–f). After 24 h, the donepezil concentration in the upper chamber reached a plateau of 3.72% of the initial quantity.

To experimentally assess the numerical model, donepezil was injected in the basal chamber of the millifluidic device and after increasing time intervals (from 1 to 72 h), the medium into the upper chamber was sampled and the drug concentration quantified as described in the Experimental Section. We found that the values of transported donepezil were coherent with those obtained from the numerical model (Figure 8f).

### Evaluation of Drug Toxicity and Metabolism in the LoC

**Figure 9:**
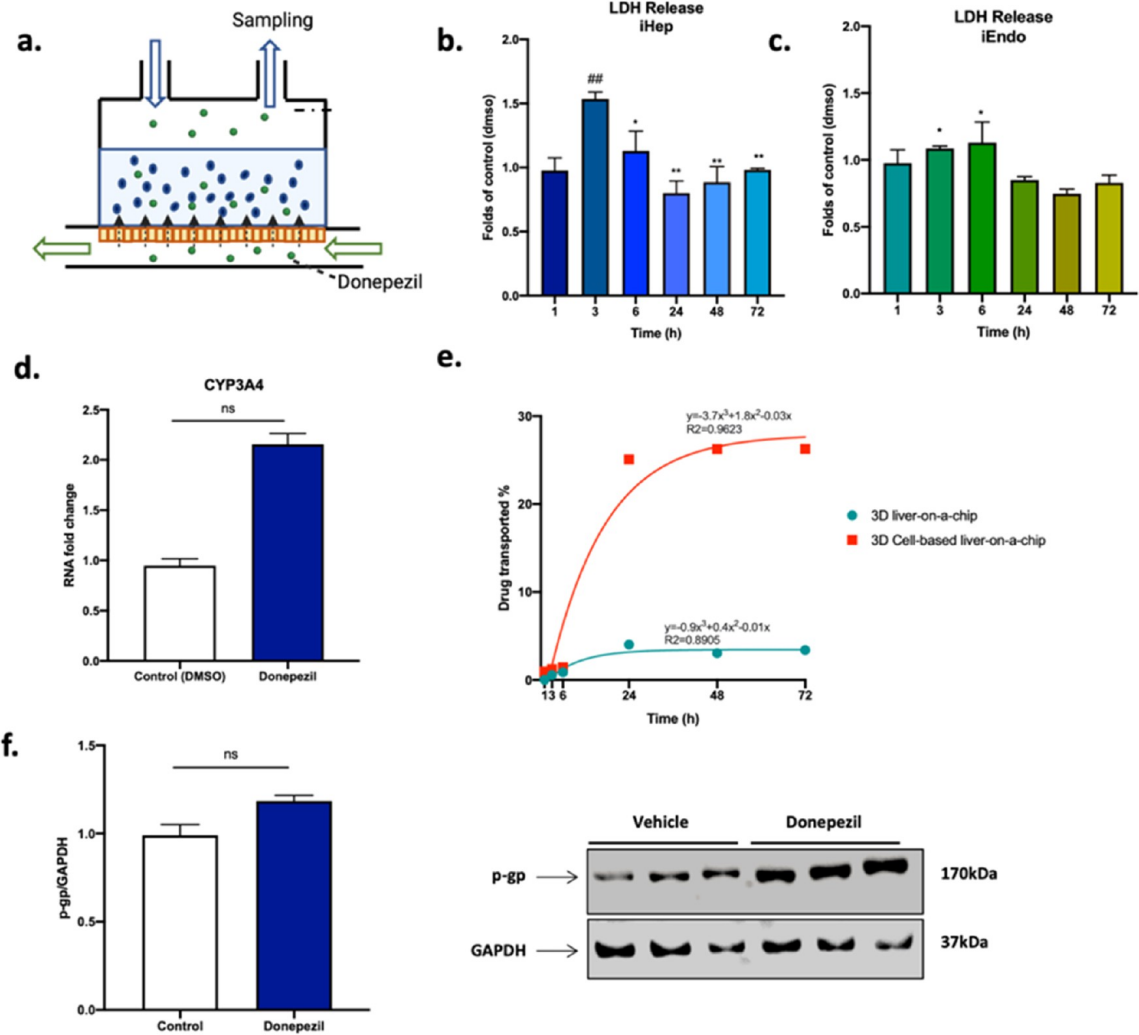
Effects of donepezil administration in the LoC. (a) Side-view schematic of the LoC in which donepezil is administrated. (b,c) LDH test of iHep and iEndo in perfusion. One-way ANOVA test and Tukey’s multiple comparison post hoc test (b) * = *p* < 0.05; ** = *p* < 0.01 vs 3 h; ## = *p* < 0.01 vs 1 h. (c) * = *p* < 0.05 vs 48 h. (d) mRNA expression of CYP3A4 of iHep in perfused samples 72 h after drug and DMSO administration. Mann–Whitney *U* test, *p* > 0.05. (e) Plot showing donepezil transport either into the 3D LoC alone or hosting cells. (f) Western blot to detect *p*-gp in the lysates of iHep in perfused samples 72 h after DMSO and drug administration. Mann–Whitney *U* test, *p* > 0.05.

The safety of 200 μM donepezil for iHep and iEndo hosted into the LoC was investigated with LDH assay at the same time intervals as before (from 1 to 72 h) after drug injection (Figure 9a). Figure 9b,c shows an increase in LDH release 3–6 h after drug injection suggestive of low cytotoxicity in the millifluidic device in both apical and basal chambers, but the trend reversed afterward. Moreover, since donepezil undergoes liver metabolism primarily through the CYP3A4 enzyme,^23^ the latter was investigated with RT-PCR 72 h after drug dosage and resulted in being higher in iHep treated with Donepezil (Figure 9d).

### Experimental Evaluation of Donepezil Transport in the LoC

Finally, the amount of drug transported through the fully assembled LoC was evaluated after 1–72 h from donepezil injection. Figure 9e reports the curve showing drug transported vs time. The amount of drug transported through the device increased from 1 to 36 h and settled to 26% at 48 h. Interestingly, the amount of drug transported into the device was higher in the 3D model in the presence of cells, suggesting the presence of a cell-mediated transport. To assess this hypothesis, the protein expression of the hepatocyte transporter *p*-gp, reported to be able to recognize donepezil as a substrate,^24−26^ was investigated. The Western blot results indicated higher values of the *p*-gp/GAPDH ratio for iHep exposed to donepezil with respect to the control condition where we administered the drug vehicle alone (DMSO) (Figure 9f).

## Discussion

In the present work, we propose an innovative LoC based on a new millifluidic OoC we developed, named MINERVA 2.0. device, hosting iHep encapsulated into a collagen–polyethylene glycol matrix^10^ and cultured interconnected with a 2D layer of iEndo to recapitulate the key features of the liver sinusoid. To assess its potential to be used for drug screening purposes, our LoC has been assessed with the AD-approved drug donepezil.^11^

MINERVA 2.0 has many key features: in our device up to 1 million cells can be hosted and perfused with up to 10 mL of culture medium, and thus, several biological tests can be performed in multiple replicate.^27^ Moreover, a great advantage of our system is due to the presence of a Transwell -like insert, which has customizable features such as different membrane materials and/or pore dimensions and densities.

In addition, the placing into the MINERVA 2.0 of the Transwell -like insert hosting the iPSC-derived cells after their maturation in the static condition avoids using enzymatic dissociation for cell harvesting from the cell culture support and re-plating inside the device, which could impair iPSC differentiation and survival. Reportable advantageous characteristics of our device are its user friendliness, optical accessibility, which allows continuous cell monitoring by microscopy, and affordability.

The peculiar design of the millifluidic MINERVA 2.0 allows also the accommodation of millimetric 3D models as hydrogels (1.5 mm thick) and the serial connection of single MINERVA 2.0 devices to build human OoC platforms.

One of the major drawbacks for the use of iHep in drug development studies is their functional immaturity when cultured alone in a standard 2D condition.^28^ Instead, it is widely accepted that a 3D microenvironment and the co-culturing with non-parenchymal cells can lead to the stabilization of the hepatocyte phenotype and functions.^29^

Thus, we evaluated the effects of iEndo and 3D culture on maturation of iHep by developing a 3D w/ iEndo model in which an endothelial layer was interconnected with a hydrogel-based 3D hepatic model. Markers of hepatocyte function, such as albumin and urea secretion and CYP3A4 expression, were significantly higher into the gel in the presence of iEndo, as reported by others.^12,30−35^ Consequently, we observed that a 3D environment and cell–cell interactions have been crucial also in our model for maintaining hepatocyte viability and function.

Thus, the 3D w/ iEndo liver model can be considered an optimized configuration to implement an LoC into the millifluidic device MINERVA 2.0.

In order to ensure the optimal oxygen supply and physiological shear stresses on the cells into the MINERVA 2.0.-based LoC, we developed a computational simulation to select the adequate perfusion rate. With a flow rate of 30 μL/min, the oxygen concentration in both chambers was in line with the in vivo values.^36^ The shear stresses were close to the physiological values for iHep, while for iEndo, they were lower.^35^ Interestingly, the presence of the hydrogel embedding the iHep acted as a barrier to the fluid flow, strongly reducing the shear stresses on the cells to values close to those in the native liver sinusoid.^37^

As for the performance of the 3D w/ iEndo liver model into the millifluidic device, the iHep resulted in being viable up to 7 days in perfusion and exhibited higher albumin and urea secretion as well as CYP3A4 basal expression with respect to the reference condition. Of notice, the albumin values of perfused iHep were lower than in vivo liver data, while the urea values were coherent.^38^ However, the values of albumin secreted by iHep are close to values of iPSC-derived hepatocytes found in the literature.^39,40^ The expression of the hepatocyte nuclear factor 4 alpha (HNF4a), a gene involved in hepatocyte differentiation, was instead comparable between dynamic and static conditions. These results suggest that globally the dynamic culture induced an increase in iHep functions and was compatible to the maintenance of the iHep mature state.

As previously stated, our LoC biologic performance took advantage of the co-culture of iEndo, which was viable up to 7 days, developed a continuous endothelial layer, and aligned along the direction of the fluid flow, a result partly unexpected due to the low shear stresses indicated by the computational analyses. This can be explained considering that several studies demonstrated the involvement not only of shear stress^41,42^ but also of other physical (e.g., stiffness and topography) and soluble factors^43,44^ in endothelial cells functioning in vivo.

Moreover, the positive labeling of the adhesion molecule CD-31, responsible for vascular differentiation, highlighted the presence of differentiated endothelial cells.^45^ We can conclude that also the endothelial cellular component of our LoC was suitable for a liver-relevant model up to 7 days in the dynamic condition.

As one main application of OoC in the field of drug development is related to liver-dependent metabolism and possible toxicity, our LoC was tested with donepezil, a drug approved by FDA for the treatment of AD.^46^

The values of drug partition and its diffusion coefficient were experimentally estimated and highlighted that the hydrogel used as a 3D matrix did not bind to the drug or hinder its passage.

Donepezil release occurred following the Fickian diffusion mechanism and release profile fitted into the Korsmeyer–Peppas model.

Through a combined computational–experimental strategy, we then quantitatively simulated the spatial and temporal gradient of donepezil into the LoC. The test ended at 72 h to meet the half-life of donepezil (70 h).^21,47^

The computational analysis confirmed that the hydrogel did not hinder the molecule diffusion. Moreover, both the strategies were concordant in estimating the concentration of donepezil at the outlet of the LoC, and it turned out that 4% of the initial quantity of the drug injected passed through the basal chamber and the hydrogel to the exit of the apical chamber. The lower amount of drug passage in the dynamic condition with respect to the static one may be due to the bifurcation of the paths of molecule diffusion in dynamics into two routes: the basal and apical chambers. Therefore, if in the static condition, the diffusion of the drug from the basal chamber can only take place toward the apical chamber; in the dynamic condition, the passage of the molecule can occur toward both the apical chamber and the basal chamber in recirculation, thus decreasing the transport of donepezil to the upper chamber. From this analysis, it is also apparent that this cell-free system requires further tuning to study donepezil kinetics if the target is an appreciable passage to the upper part of the device.

However, this limitation was partly overcome when donepezil was tested at a concentration of 200 μM into the LoC hosting both iHep and iEndo cells. The toxicity profile and metabolic effect of the drug on the cells were satisfactory, and we recorded as the main effect that the amount of drug transported was greatly increased, more than 5 times than that obtained in the LoC without cells. We also observed an increasing trend of expression levels of CYP3A4 in iHep treated with the compound.

This cell-mediated effect likely relies on an increase of active transport. In fact, donepezil was confirmed as a substrate of the hepatic transporter *p*-gp whose expression is increased by stresses as exposure to xenobiotics.^23,24,46^ Coherently, the *p*-gp protein expression of iHep in our LoC in the presence of donepezil was higher, corroborating our hypothesis.

To the best of our knowledge, the LoC presented in this work is the first LoC which integrates a co-culture of iPSC-derived liver cells with an endothelial layer interconnected with a 3D hydrogel-based hepatocyte model. In fact, few iPSC-based LoCs developed so far are populated either by iHep only^48−50^ or by iHep and non-parenchymal cells from human cell lines.^51,52^ Only few studies focused on integrating a hydrogel in the LoC,^53^ and there is only one system hosting iHep encapsulated in a hydrogel^49^ in which cell survival and albumin secretion are lower if compared to our results.

At present, our model features hepatocytes and endothelial cells but may be improved by adding important resident cells of the sinusoids such as Kupffer cells and hepatic stellate cells. Furthermore, in this work, we used Transwell -like inserts hosting PET membranes that are a common component of OoC devices, as reported by Salimbeigi et al., 2022.^54^ These membranes are standardized but do not fully replicate the Space of Disse basement membrane nano-fibrillar architecture and are characterized by lack of biochemical cues and higher stiffness and thickness (30 μm in comparison to 1.4 μm) if compared to Space of Disse.^55^ The potential use of biomimetic ad hoc tailored membranes might help in better representing the biological cues.

## Conclusions

We have reported a novel LoC, based on our new millifluidic OoC device MINERVA 2.0, integrating a co-culture of iPSC-derived liver and endothelial cells and a hydrogel-based 3D model, having a millifluidic scale and suitable to support dynamic culturing of human iPSC-derived cells recapitulating key biological features of liver physiology.

Current in vitro assays for hepatotoxicity testing are based on primary human hepatocytes, which rapidly lose their polarity and functionality in vitro and suffer from high donor-to-donor variability and cell lines such as HepG2, with have a lower metabolic capacity with respect to primary hepatocytes. Differently, iHep have emerged as advantageous for modeling liver functions since among their benefits, there are preserved differentiation and physiological functions.

2D liver models allow for a prolonged culture, but they are poor representative of relevant physiological cues as fluid shear stress and three-dimensional cell–cell interactions, while 3D culture models such as hydrogels and flow-based culture as OoC have shown to improve hepatocyte functionality and maturity.

Another remarkable feature of our LoC is that, thanks to the MINERVA 2.0 device, it can be serially connected upstream and downstream to other basic units, resulting in multi-organ platforms. The exposure to donepezil demonstrated a biological modulation of the system, making it suitable for drug development purposes, also from the perspective of iPSC-based personalized medicine.

## Supporting Information Available

Numerical values and parameters used for the simulations and TEER assessment (PDF)
