# Supplementary material for: Development of an Induced Pluripotent Stem Cell-Based Liver-on-a-Chip Assessed with an Alzheimer’s Disease Drug
Source: ACS Biomater Sci Eng. 2023 Jun 15;9(7):4415–30. doi: 10.1021/acsbiomaterials.3c00346 (PMC10336847; doi:10.1021/acsbiomaterials.3c00346)
Supplement: Supplementary file 1 — ab3c00346_si_001.pdf [file ab3c00346_si_001.pdf]

Supplementary material

Development of an induced pluripotent stem cells-based liver-on-a-chip assessed with an Alzheimer's disease drug.

F. Fanizza[1], L. Boeri[1], F. Donnaloja[1], S. Perottoni[1], G. Forloni[2], C. Giordano[1*], D. Albani[2]

1. Department of Chemistry, Materials and Chemical Engineering 'Giulio Natta', Politecnico di Milano, Milan, Italy.

2. Department of Neuroscience, Istituto di Ricerche Farmacologiche Mario Negri IRCCS, Milan, Italy.

[*] Corresponding author

Carmen Giordano

carmen.giordano@polimi.it

SUPPLEMENTARY TABLES

Table S1 Numerical values of the parameters used for the simulations.

Property	Value
Atmospheric pressure (outlet)	0 Pa
Wall conditions	No slip conditions
Density of culture medium (at 37°C)	1000 kg/m^3 [42]
Dynamic viscosity of culture medium (at 37°C)	$8.1*10^{-4}$ Pa*s [42]
Diffusion coefficient of oxygen	$1.85*10^{-10}$ cm^2/s
Pump flow rate	30 µl/min

Inlet velocity in the upper chamber	0.001 m/s
Inlet velocity in the lower chamber	0.19*10^{-3} m/s
Membrane permeability	7.8*10^{-19} m^2
Membrane porosity	2.5*10^{-3}
Oxygen concentration at the inlets	0.2 mol/m^3
Density of COLL-PEG2000 hydrogel (at 37°C)	1000 kg/m^3
Dynamic viscosity of COLL-PEG2000 hydrogel (at 37°C)	8.1*10^{-4} Pa*s
COLL-PEG2000 hydrogel permeability	6.45*10^{-17} m^2
COLL-PEG2000 hydrogel porosity	0.93
iPSC-derived hepatocytes oxygen consumption rate	4.5 nmol/(min*10^6 cell) [20]
iPSC-derived endothelial cells oxygen consumption rate	1.5 pmol/min/1000 cells [21]

Table S2 Numerical values of the parameters used for the donepezil distribution simulations.

Property	Value
COLL-PEG2000 hydrogel permeability to donepezil	6.45*10^{-17} m^2
Donepezil diffusion coefficient in iEndo culture medium	4,16 x $10^{-11.}$ m^2/s
Donepezil diffusion coefficient in iHep culture medium	4,12*10^{-11} m^2/s
Donepezil diffusion coefficient through the hydrogel	7,2772 x $10^{-12.}$ m^2/s

Donepezil concentration at the inlet	0.2 mol/m^3
Donepezil partition coefficient	1.07

SUPPLEMENTARY EXPERIMENTAL

Transepithelial electrical resistance assessment

Transepithelial electrical resistance (TEER) was measured using EVOM (World Precision Instruments, USA) coupled with a chopstick-like electrode. Cell layer resistance was calculated placing the shorter electrode in the apical compartment of the Transwell®-like inserts and the longer one in contact with the plate. TEER ($\Omega \cdot cm^2$) was calculated as follows:

$$TEER = (Rmeasured - Rblank) \cdot MembraneArea$$

Where Rblank was measured on Transwell®-like inserts without cells and the $MembraneArea$ was 1.13 cm^2. For each sample, we averaged three measures.

SUPPLEMENTARY FIGURES

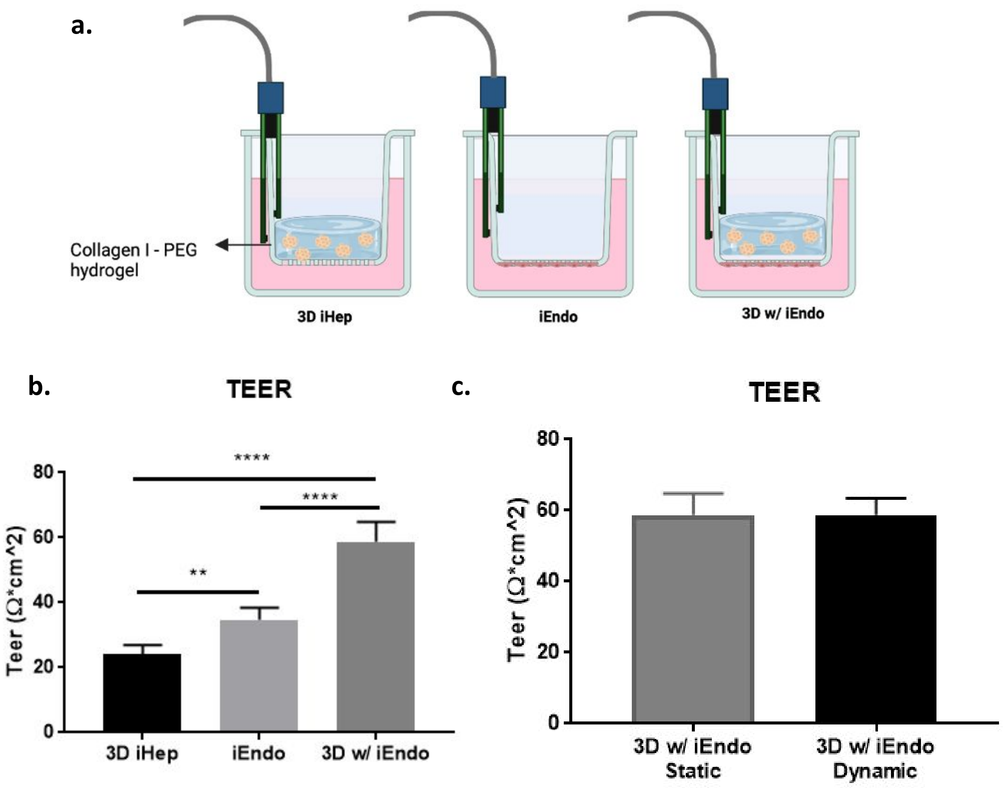

Figure S1. a) The schematic shows the cell models assessed with the TEER test into the Transwell®-like insert. b) For each model we measure TEER in static condition. One-way ANOVA, Tukey's multiple comparison post hoc test **=p<0.01; ****= p<0.0001. c) TEER values of the 3D w/ iEndo model in static and dynamic condition. t-test p>0.5.
